# Hierarchically Porous Anatase Nanoparticles Derived from One-Dimensional Lepidocrocite Titanate for Bisphenol-A Photodegradation

**DOI:** 10.1021/acsomega.4c07224

**Published:** 2024-12-11

**Authors:** Treesa Reji, Adam D. Walter, Yasunori Hioki, Tracey Curran, Mary Qin Hassig, Hussein O. Badr, Gregory R. Schwenk, Takeshi Torita, Megan A. Creighton, Michel W. Barsoum

**Affiliations:** † Department of Materials Science and Engineering, Drexel University, 3141 Chestnut Street, Philadelphia, Pennsylvania 19104, United States; ‡ MuRata Manufacturing Co., Ltd., 10-1 Higashikotari 1-chome, Nagaokakyo-shi, Kyoto 617-8555, Japan; § Academy of Natural Sciences of Drexel University, 1900 Benjamin Franklin Pkwy, Philadelphia, Pennsylvania 19103, United States; ∥ Department of Chemical and Biological Engineering, Drexel University, 3141 Chestnut Street, Philadelphia, Pennsylvania 19104, United States

## Abstract

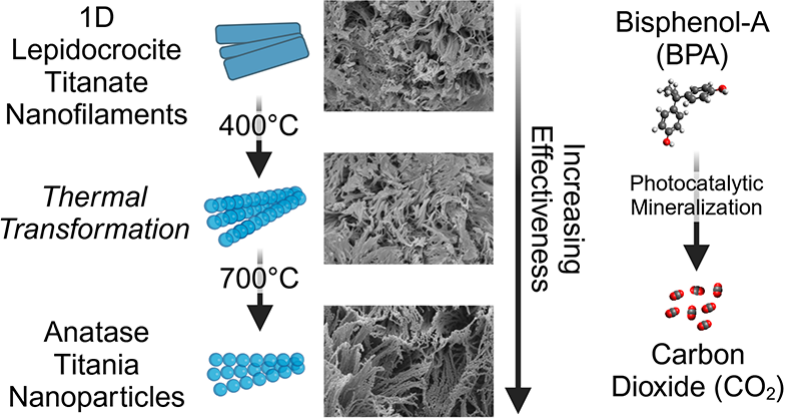

Herein, we discuss the conversion of one-dimensional lepidocrocite (1DL) titanate nanofilaments to anatase. Upon heating at temperatures >400 °C, the hierarchical 1DLs porous mesostructured particles transform to anatase, while retaining their morphology. These assemblies are characterized via X-ray diffraction, scanning and transmission electron microscopy, Fourier transform infrared spectroscopy, and solid-state ultraviolet absorbance. The assemblies were tested in the photodegradation of a water-soluble, endocrine-disrupting organic compound, bisphenol A (BPA). Using ultraviolet–visible spectroscopy, we show that 95% of BPA is degraded in 1 h under 1 sun of the simulated solar spectrum. Under the same conditions, the total organic carbon of the solution was reduced by 70%.

## 1 Introduction

Nanostructured titanium oxides have been, and remain, of significant interest due to their enhanced properties such as greater surface area, enhanced reactivity, and unique electrical characteristics. They have been explored in diverse applications across various scientific and technological fields, such as photocatalysis,^1^ photovoltaics,^2^ self-cleaning films,^3^ sensors,^4^ air purification,^5^ and biomedicine,^6^ among others. Comprehending, and controlling, the morphology of nanoparticles (NPs) are essential for customizing products to fulfill certain demands of the aforementioned applications.^7^

Titanium dioxide (TiO_2_), or titania, is available in different structural polymorphs: anatase, rutile, and brookite.^8^ The crystal structures of these polymorphs differ in terms of the arrangement of the TiO_6_ octahedra. More oxygen-rich titanates are available in different structures such as spinel,^9^ perovskite,^10^ and, most related to this work, two-dimensional (2D) layered lepidocrocite-type structures.^11^ To neutralize the titanate crystal, cations are required and their locations vary based on the product.^12,13^

In terms of morphology, dimensionality is of major importance. One-dimensional (1D) and 2D materials exhibit properties that are distinct from their three-dimensional counterparts. 1D titania nanostructures have received some attention for their high aspect ratios, high specific surface areas (SSAs),^14,15^ and confinement of charge carriers along one dimension.^16^ The 1D morphology has also been conjectured to reduce charge recombination.^17^ The large SSAs render them promising candidates in nanodevices,^18^ water splitting,^19−21^ photocatalysis,^22−25^ and removal of organic pollutants,^26−28^ the subject of the work presented herein. 1D TiO_2_ and titanate nanostructures with various morphologies such as nanotubes,^29−31^ nanorods,^32^ nanowires,^33,34^ nanofibers,^35,36^ nanosheets,^11,37−40^ and nanobelts^41,42^ have been reported.^43−47^ These exhibit many characteristic properties of titania NPs and can possess substantial SSAs (≈200 m^2^ g^–1^) which can contribute to a higher density of active catalytic or adsorptive sites.^13,48−50^

Various experimental factors affect the crystal phases of nanostructured titania, including synthesis method, annealing temperatures, and synthesis duration, which may impact their photocatalytic performance.^51,52^ One of the most often utilized semiconductor photocatalysts is anatase,^53^ which outperforms rutile in certain applications.^54−63^ Anatase is thought to have more reactive surface sites, which may facilitate adsorption and reactions with molecules, thus, it is employed as a photocatalyst in a number of environmental remediation processes, such as the degradation of different organic pollutants.^58,64−66^ Various structural and morphological modifications of TiO_2_ can vastly impact photocatalytic performance.^52,67−69^

A novel titanate, recently discovered by our group, provides an inexpensive and vastly scalable method to prepare 1D titanates. The synthesis of these low-dimensional materials (base-unit cross section of just ≈ 6×6 Å^2^) relies on tetramethylammonium hydroxide (TMAOH) as a solvating and templating agent for a wide array of Ti-containing precursors. TMAOH appears to be the key to the synthesis procedure–when using alkaline bases,^70^ instead, we obtain alkali titanates similar to those reported in the literature.^71^ These nanofilaments (NFs), henceforth referred to as 1D lepidocrocite (1DLs), grow in the *a* direction and, upon drying, stack along the *b* direction in the plane to form ribbons or 2D flakes.^72^ They are confined in the *c* direction, making up the cross section. 1DLs easily form aqueous colloidal suspensions^73^ and self-assembled films^74^ or can be fabricated into free-flowing porous mesostructured particles (PMPs)^75,76^ that are the subject of this work.

With regard to applications, 1DLs excel in applications where surface area is important. They adsorb sulfur^77^ and inorganics.^75,78^ Colloidal 1DLs (1DL-Col) have demonstrated effective adsorption, and subsequent photodegradation, of common organic dye pollutants, such as rhodamine 6G and crystal violet^79^ and in more recent work malachite green.^80^ These dyes also sensitize the 1DLs that allows for their degradation under visible light only. To date, the adsorption and degradation of organic molecules by 1DL PMPs has yet to be reported in the literature.

Titania-based nanomaterials have been widely studied to degrade endocrine-disrupting compounds (EDCs).^81^ By definition, EDCs are a serious concern to the aquatic environment since they are often found in wastewater and cause hormonal disorders in multicellular creatures.^82,83^ An endocrine disruptor, bisphenol A (BPA), has been linked to impacts on thyroid and estrogenic hormones.^84^ BPA is widely employed in the production of chemical products, like epoxy resins, plasticizers, flame retardants, and polycarbonate plastic.^85,86^ It is water-soluble, toxic, and resistant to biodegradation,^84^ and its presence in industrial and domestic water sources has been reported.^88^ Conventional water treatment facilities, however, are inadequate to handle this kind of pollution due to typically high energy consumption and incomplete removal and formation of toxic byproducts.^89^ TiO_2_ has been shown to degrade EDCs, like BPA, under relatively mild conditions, in particular Aeroxide P25.^90−93^

The purpose of this work was to explore the use of 1DLs for degrading EDCs, like BPA. We show that untreated 1DL PMPs did not photodegrade BPA. However, once annealed at various temperatures, 1DL PMPs maintain their microscale morphology, but on the nanoscale, they partially spheroidize and undergo a structural transformation into anatase (AN). These anatase hierarchical assemblies were then employed to study the photodegradation of BPA in water and were compared to commercial TiO_2_ nanomaterials. The kinetics of degradation were studied using ultraviolet–visible (UV–vis) spectroscopy, and the extent of mineralization was confirmed by total organic carbon (TOC) analysis. The experimental data obtained fit well with a zero-order kinetic model. Limited research is available for BPA degradation following the zero-order model which correlates the degradation rate with the activity of the catalyst rather than the reactant concentration.^94,95^ Overall, this study provides insight into the thermal stability of 1DLs and explores the structural changes associated with annealing.

## 2 Results and Discussion

### 2.1 Structural Characterization

**Figure 1:**
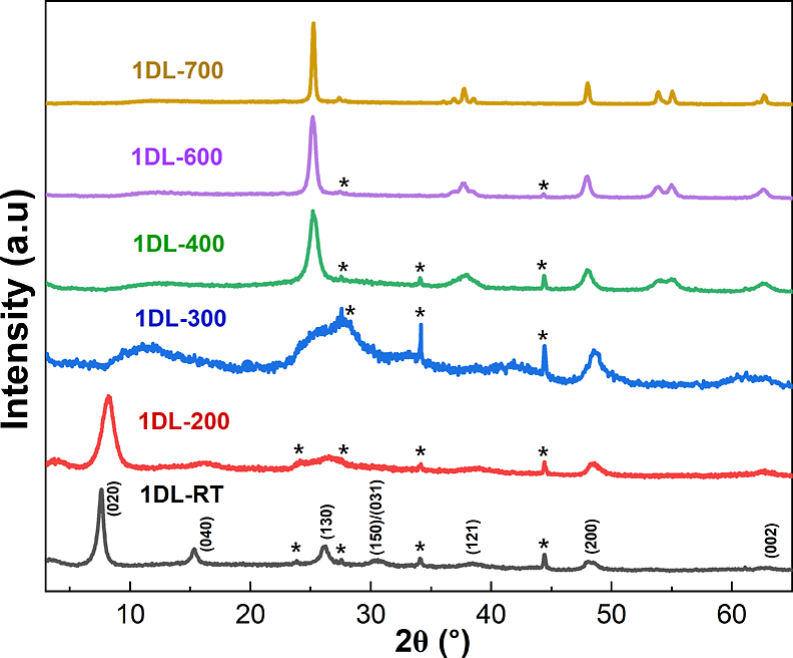
XRD patterns for 1DL powders as-synthesized and after heating in the 200 to 700 °C range in open air for 12 h. Labels and patterns are color-coordinated. 1DL powders were prepared by shaking TiB_2_ in a TMAOH aqueous solution at 80 °C for 5 d, rinsed with ethanol, and dehydrated at 50 °C in air before further processing. Asterisks denote peaks corresponding to the unreacted TiB_2_ precursor.

All sample designations are available in Table 1. As discussed in the experimental details section, the sample designations were 1DL-xx, where xx is RT for room temperature or the annealing temperature. Another set of samples is labelled AN-xx for a commercially obtained anatase-based material. We also tested as-received P25, a nanosized titania made by Evonik. The 1DL-to-anatase transformation is evident from the X-ray diffraction (XRD) patterns shown in Figure 1. 1DL PMPs (black pattern, Figure 1) show a pattern consistent with prior work, primarily peaks at 2θ ≈ 8° (020), 48° (200), and 62° (002) that are consistent with lepidocrocite.^75^

After being heated to 200 °C, the 1DL-200 samples maintained the 1DL structure (red pattern, Figure 1), but the 0*k*0 peak was wider. The 1DL-300 pattern (blue pattern,Figure 1) is quite ill-formed and is, most probably, a mixture of anatase and lepidocrocite phases, where the transformation to the anatase phase begins due to TMA^+^ decomposition. By 400 °C, the transformation to anatase is complete (top 3 patterns, Figure 1). The peaks and their indices obtained at these annealing temperatures are 2θ ≈ 25.1° (101), 37.8° (004), 48.1° (200), 53.9° (105), 55° (201), and 62.6° (213) and correspond to anatase (JCPDS CAS no. 21-1272).

To better understand the characteristics of our anatase, we compared our patterns with those obtained when the AN powder was heated to 12 h at the same temperatures as those of the 1DLs. From the results, shown in Figure S2, we conclude that in all cases, the resulting material is anatase.

When the domain sizes along [200] for these samples are compared (Table 1), we conclude that they all fall in the relatively narrow range of 7 to 25 nm. Not surprisingly, the domain sizes increase with annealing temperatures. However, the growth of the 1DLs with temperature (7 to 27 nm) is faster than that of the AN or P25 powders (compare 1DL with AN and P25 in Table 1). This comment notwithstanding, when the 1DL and P25 XRD patterns are compared after annealing at 700 and 800 °C, respectively (Figure S3), there is little doubt that the 1DL anatase is considerably more resistant to transforming to rutile than P25.^13^ This is a somewhat surprising result because the anatase-to-rutile transformation usually occurs at temperatures <800 °C.^13^ The transformation temperature also tends to reduce with decreasing particle size,^58,96^ rather than with their increase. The 1DLs powders heated to 200 and 300 °C were the least crystalline (red and blue patterns in Figure 1).

**Table 1:** Sample Designation and Annealing Temperatures Used Herein^a^

sample designation	temperature, °C	domain size, nm	domain size TEM (nm)
1DL-RT	room temperature, RT	7.0	
1DL-200	200	6.8	
1DL-300	300	5.7	
1DL-400	400	9.4	
1DL-600	600	14.5	
1DL-700	700	26.6	40.4
AN-RT	as received, RT	10.4	
AN-400	400	10.8	
AN-600	600	12.3	
AN-700	700	18.0	
P25-RT	as received, RT	25.5	
P25–400	400	17.7	
P25–700	700	23.9	

a In all cases, the annealing time was 12 h. The third column reports the domain size calculated from FWHM of (200) peaks in XRD patterns using the Scherrer formula. The last column reports the domain size estimated from TEM micrographs.

**Figure 2:**
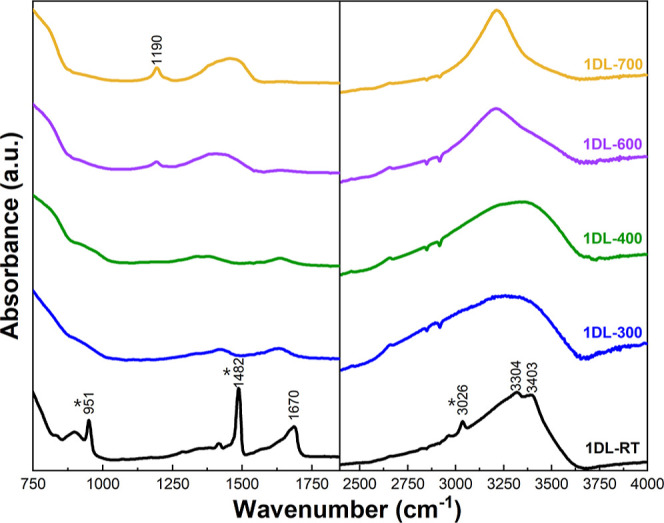
FTIR spectra of samples as a function of annealing temperatures. From bottom to top: 1DL-RT, 1DL-300, 1DL-400, 1DL-600, and 1DL-700. Labels and spectra are color-coordinated. * Indicate peaks associated with TMA^+^.

The typical Fourier transform infrared (FTIR) spectrum of the 1DL-RT sample (bottom black, Figure 2) confirms the presence of TMA^+^, with peaks at 951 cm^–1^, 1482 cm^–1^, and 3026 cm^–1^.^88^ The peaks at ∼1670 cm^–1^ and ≈3400 cm^–1^ correspond to the bending and stretching of H_2_O, consistent with other lepidocrocite-structured layered titanates.^93^ Once heated to 300 °C (blue curve, Figure 2), the 1DLs begin to dehydrate, as illustrated by the reduction in the peak intensity at ∼1670 cm^–1^, and the TMA^+^ begins to break down, as evidenced by the loss of the 951 cm^–1^, 1482 cm^–1^, and 3026 cm^–1^ peaks. However, some –OH character remains as evidenced by peaks in both the 3000 cm^–1^ to 3500 cm^–1^ and the 1300 cm^–1^ to 1500 cm^–1^ ranges, consistent with hydrated minerals and nano-titania.^94,95^ As the samples are heated further (purple and yellow curves, Figure 2), a peak at ∼1190 cm^–1^ arises that can be ascribed to Ti–O–Ti vibrations.^96^

X-ray photoelectron spectroscopy (XPS) was conducted on the 1DL-anatase powders to understand the oxidation behavior as a function of the annealing temperature. It is consistent with the FTIR (Figure 2) that there is a significant reduction in the N signal (Figure S4), indicative of the loss of TMA^+^. There is also a shift in the Ti 3/2 2p peak to higher binding energy (B.E.) (Figure S5) as temperature increases, which is most likely due to an increase in the oxidation state of Ti.^97^ The O 1s spectra show that there is a loss of metal hydroxide character (B.E. ≈ 532 eV)^98^ and the appearance of a peak at ≈533 eV in 1DL-700. This peak at ≈533 eV may be related to the shift seen in the B 1s spectra, corresponding to the emergence of further oxidized B species, such as borates.^99^

### 2.2 Morphological Characterization

**Figure 3:**
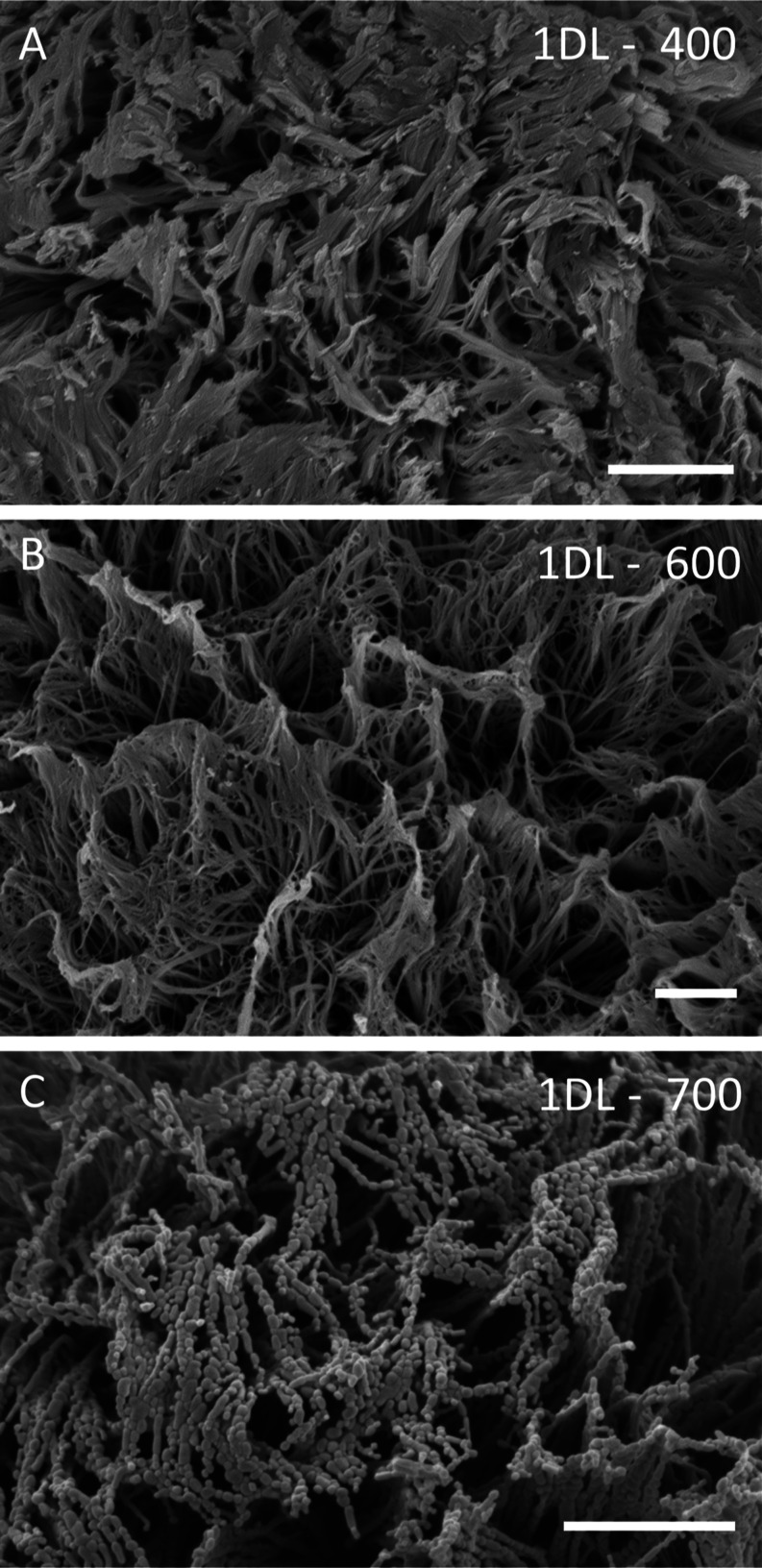
SEM micrographs of 1DL PMPs after annealing for 12 h at, (A) 400, (B) 600 and (C) 700 °C. Marker corresponds to 1 μm. Additional SEM micrographs available in Figures S6 and S7.

**Figure 4:**
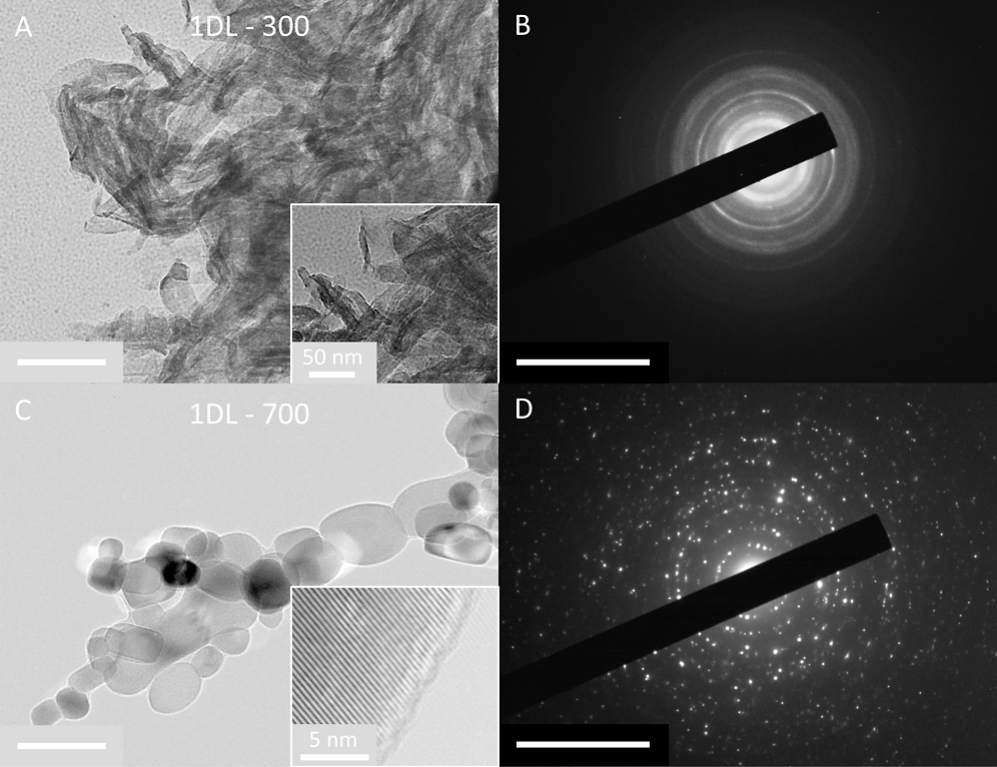
TEM micrographs and SAED patterns from samples, (A,B): 1DL-300 and (C,D) 1DL-700. Scale bars for A and C, 100 nm. Scale bars for B, D: 10 nm^–1^. Insets depict high-magnification images. Additional TEM micrographs are shown in Figure S8.

Figure S6 and Figure S7 show a collection of scanning electron microscope (SEM) micrographs, at various magnifications, of most 1DL samples fabricated and tested herein. All samples depict the PMP morphology (Figures S6A, column i and S7) that is identical to the one we reported on earlier.^78^ At low magnifications, columns i and ii in Figure S6, it is obvious that annealing, even at 700 °C for 12 h, does not change the overall PMP morphology. It is only at the highest magnification micrographs and after annealing at 600 °C, or 700 °C, for 12 h, shown in Figure 3B,C, respectively, that the otherwise smooth 1DL microfilaments roughen up when they transform to anatase. The NPs appear as near-ellipsoidal beads on a long chain suggesting that the phase transformation is accompanied by, or causes, a Raleigh instability. These ellipsoidal NPs are also shown in transmission electron microscope (TEM) micrographs (Figure 4). This result is again of utmost importance because we now have a microscale structure, PMPs, comprised of anatase NPs of the order of ≈10 to 27 nm depending on annealing temperature. Said otherwise, we now can mass-produce nonagglomerating, anatase NPs at the kilogram, or more, scales. Note that annealing at 400 °C does not alter the smoothness of the original 1DL structure even though it is now composed of anatase NPs (Figure 3A).

When the PMPs form, they range in size (Figure S7A). Their particle size distributions are shown in Figure S7 and appear to be weak functions of annealing temperatures. This is an important result because it shows that the PMPs sizes and particle size distributions are stable to 700 °C. Note that in some cases, large agglomerates form (center in Figure S7B) that were not included in the particle size distribution and are present. Otherwise, the average sizes of the PMPs all hover around 10 μm.

**Figure 5:**
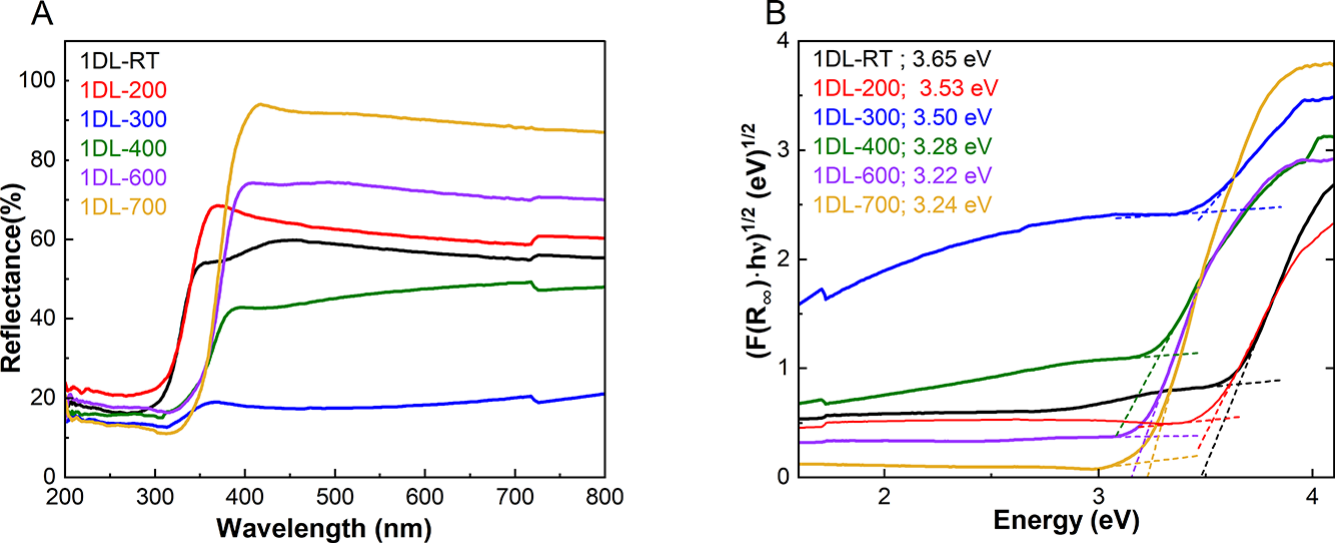
Optical properties. (A) UV–vis reflectance spectra of 1DL samples indicated on the plot. (B) Tauc plot of the same results assuming an indirect transition. In both plots, sample designation, their *E*_g_ and curves are color-coded.

Insets in Figure S6i (left column) are photographs of the powders after annealing. Increasing the annealing temperatures results in a gradual bleaching of the powders, which is also reflected in the UV–vis results (Figure 5).

TEM micrographs offer further insight into the morphological and structural changes in the samples. At low magnifications, the PMPs are easily visible (Figure S8D). Here, due to the thickness of the PMPs, only edge regions could be imaged in the TEM. Micrographs of the 1DL-RT sample (Figure S8A) show filaments, consistent with our previous work.^72^ The arcs shown in the selected area electron diffraction (SAED) pattern (Figure S8B) are irrefutable evidence for the 1D nature of the NFs comprising the PMPs.^76^

The 1DL-300 (Figures 4A,B and S8C) samples show some spheroidization and structural amorphization, where rings instead of arcs in the SAED patterns are observed (Figure 4B). Morphologically, this sample is unique in that the shapes of the individual particles seem to be between 1D filaments observed at RT and anatase NPs observed after higher annealing temperatures. At a lower magnification (Figure S6D), they appear to be microscale spheres.

The 1DL-700 samples illustrate near-spheroidization at high magnification (Figures 4C and S8F), primarily showing ellipsoid-like NPs. At lower magnification, the filamentous morphology can still be easily seen (Figure S8E), consistent with the SEM images (Figure 3C). The structural change to polycrystalline NPs is also apparent in the SAED pattern (Figure 4D). A high-resolution TEM image (inset in Figure 4C) images lattice fringes with the distance between them measuring ∼3.7 Å, consistent with the *a*-lattice parameter of anatase. This is also consistent with the XRD pattern of the 1DL-700 samples (yellow pattern, Figure 1).

### 2.3 Optical Properties

Solid-state UV–vis reflectance spectra on all 1DL samples tested are shown in Figure 5A. Figure 5B plots the corresponding Tauc plots, assuming the materials are indirect semiconductors. The results for the AN and P25 powders are shown in Figure S9B, as a function of annealing temperatures; the corresponding Tauc plots are shown in Figure S9C. Table 2 summarizes the band gap and *E*_g_ values. In short, all *E*_g_ values for the anatase samples fall in the narrow range of 3.25 to 3.27 eV and are thus in line with other published results.^100−102^ The value for P25 is the lowest, presumably because it contains rutile. The *E*_g_ for the 1DL-RT sample is the highest, 3.65 eV; the *E*_g_ values for the 1DL-200 and 1DL-300 fall in between those of 1DL-RT and anatase.

More specifically, it is well-established–both theoretically^103^ and experimentally—that anatase has an indirect *E*_g_ of 3.25 eV.^102,104,105^ The situation for lepidocrocite is less clear currently. Density functional theory calculations on 2D lepidocrocite predict a direct band gap,^106^ while most of the experimental work, for reasons that are unclear, assumes it to be indirect.^107^ Until this is resolved, we assume both: indirect in Figure 5B and direct in Figure S9 and plot the Tauc plot accordingly. This comment notwithstanding, we are inclined to assume lepidocrocite titanates have direct band gaps. Based on the results shown in Figures 5 and S9 and Table 2, we conclude that in accordance with all our work to date, at 3.65 eV, *E*_g_ of the 1DL-RT (black line, Figure 5B)—assuming indirect—is substantially higher than all other samples. If one assumes a direct transition (Figure S7A), the discrepancy is even larger. This enhancement is due to quantum confinement.^102^ With increasing annealing temperatures, the *E*_g_ values decrease to 3.5 eV at 300 °C and asymptote to ≈3.3 eV, by 400 °C (Table 2). Higher reflectance of the 1DL-700 sample (orange line, Figure 5A) is attributed to an improvement in crystallinity. For reference, the indirect *E*_g_ value for a drop-cast 1DL-Col measured previously is ≈ 4 eV.^108^ The discrepancy from this value and the 3.65 eV reported here is probably due to the self-assembly of the 1DLs into 2D or pseudo-2D morphologies, in which case the quantum confinement is only in one dimension.^37^ The results for the AN and P25 samples shown in Figure S9B,C are in line with published results.^104^*E*_g_ for anatase is ≈ 3.25 eV^102^ and that for P25 is closer to 3 eV.^104,109^

The Urbach tail absorption is maximum for the 1DL-300 samples (blue line, Figure 5A,B); the second highest is 1DL-400 (green line, Figure 5A,B), followed by 1DL-RT (black line, Figure 5A). The background drops further after the 600 °C and 700 °C anneals. If the plausible assumption is made that these tails are due to defects, the results make sense as follows. As-received 1DL-RT samples (black curve, Figure 5B) contained some defects. The defect concentrations increase dramatically after the 300 °C annealing (blue curve, Figure 5B) and then monotonically decrease as the annealing temperatures increase to 700 °C, where the Urbach tails almost vanish. The AN-RT and P25-RT samples (Figure S9B,C) show sharp absorption onsets and a decrease in the Urbach scattering that indicate more-ordered, less-defective, structures. At this juncture, the nature of these defects is unknown and outside the scope of this paper. The 1DL-300 (blue curve in Figure S9A) sample is the least reflective, which explains why it is the darkest sample (inset in Figure S6 B, column i). The most likely reason for this is the presence of C from the incomplete decomposition of the TMA^+^ cations present after synthesis.

**Table 2:** Indirect and Direct Energy Band Gap Values of Various Samples

samples	*E*_g_ (eV) (indirect)	*E*_g_ (eV) (direct)
1DL-RT	3.65	3.80
1DL-200	3.53	3.72
1DL-300	3.50	3.39
1DL-400	3.28	
1DL-600	3.22	
1DL-700	3.24	
AN-RT	3.25	3.42
AN-400	3.27	
AN-600	3.25	
AN-700	3.26	
P25-RT	3.05	

### 2.4 Degradation of BPA

**Figure 6:**
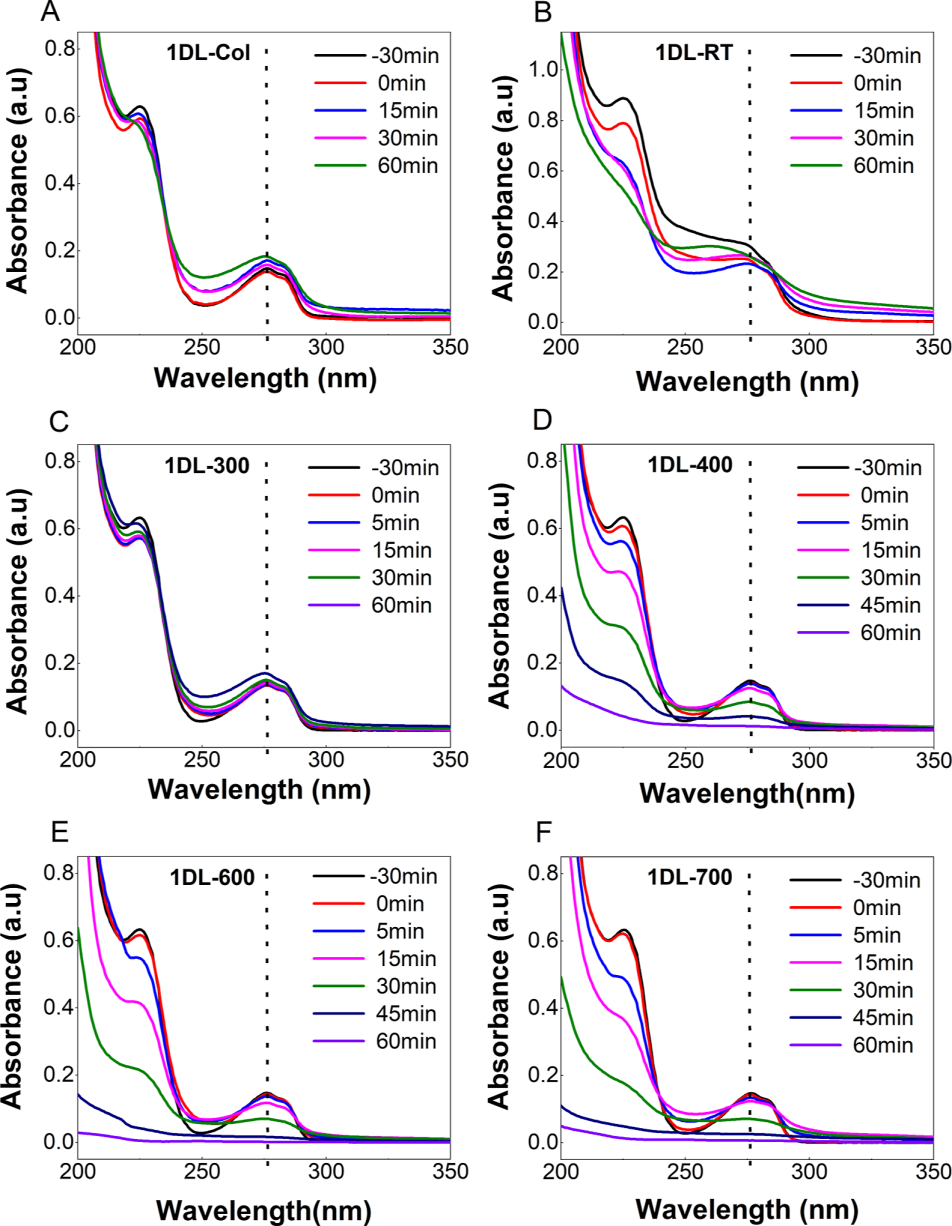
Time dependence of BPA degradation at a concentration of 10 mg/L by, (A)1DL-Col(B) 1DL-RT, (C) 1DL-300, (D) 1DL-400, (E) 1DL-600, and (F) 1DL-700. Vertical dashed lines correspond to λ = 276 nm which is wavelength used to follow BPA degradation. The BPA-to-1DL molar concentration was 1:1 in all cases.

**Figure 7:**
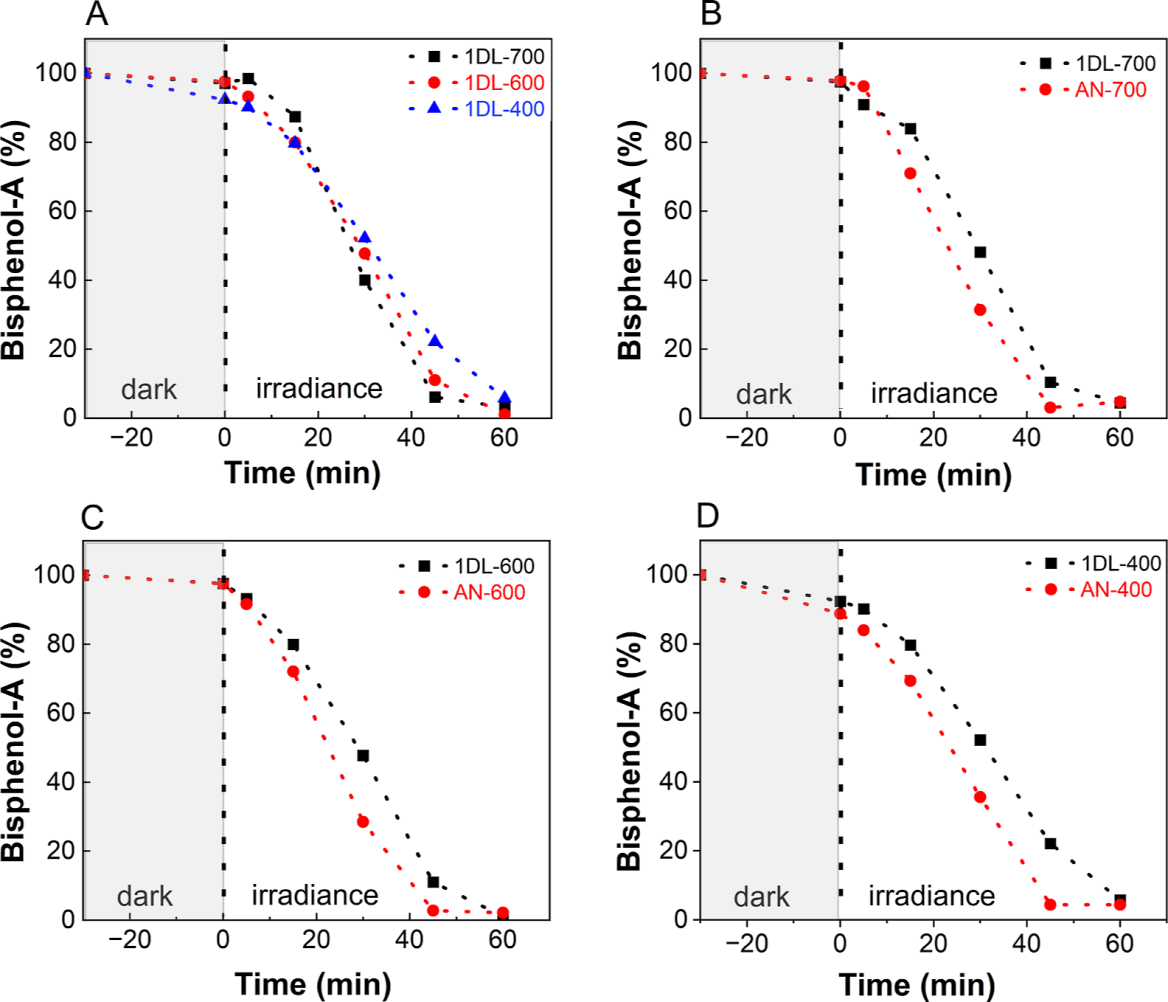
Time dependence of BPA degradation over 1DL-derived anatase curves for photocatalytic oxidation of BPA degraded by, (A) 1DL-700, 1DL-600, and 1DL-400, (B) 1DL-700 and AN-700, (C) 1DL-600 and AN-600, and (D) 1DL-400 and AN-400. Monitored by UV–vis at 276 nm. Labels and curves are color coordinated.

Typical absorbance spectra for 6 different 1DL samples—1DL-Col, 1DL-RT, 1DL-300, 1DL-400, 1DL-600, and 1DL-700—mixed with BPA are shown in Figure 6A–F, respectively. Figure 7 plots the same results after converting them to a normalized BPA concentrations, assuming that the Beer–Lambert law applies. Figure S10 compares all of the materials tested.

BPA has absorption peaks at 276 and 225 nm, and either, or both, can be used to follow its degradation. Said otherwise, the intensity of both excitation peaks must be reduced to claim the removal of aromaticity of BPA.^110^ Since most researchers in this area use λ between 270 and 280 nm to determine the BPA concentrations, we do the same.^111^ Note that both peaks are reduced with time.

**Figure 8:**
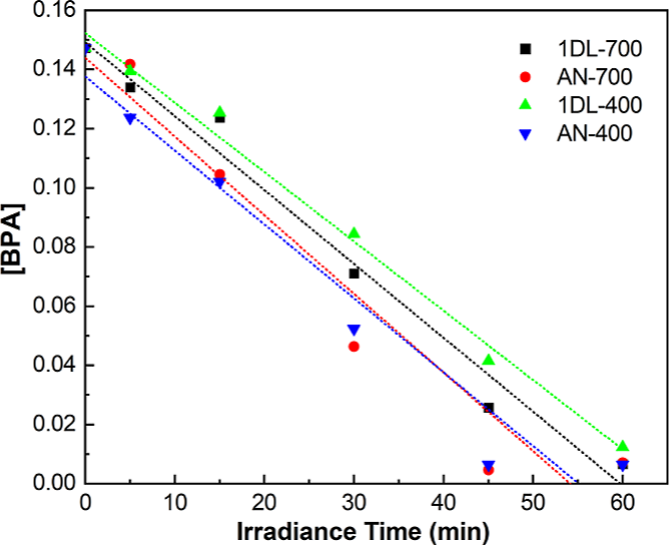
Linear fit between BPA concentration and reaction time for select samples after 12 h annealing at temperatures indicated. Sample designation and lines are color-coded. Kinetic data is listed in Table 3. Labels and curves are color coded.

To shed more light on the degradation mechanisms, we plotted the time dependencies of the BPA concentrations assuming a zero-order kinetic model, viz., eq 1, below
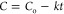
1where *k* is the rate constant, *C*_0_ is the initial BPA concentration, and *C* is the concentration at time *t*.^112^ Note *C*_o_ is the concentration at *t* = 0, after the hold in the dark for 30 min and not the initial before, viz., 10 g L^–1^. Table 3 summarizes the kinetic data, and Figure 8 plots the time dependency of the BPA concentrations as a function of catalyst used.

**Table 3:** Summary of Kinetic Parameters of BPA Degradation by 1DL-400, 1DL-700, AN-400, and AN-700 Powders^a^

samples	extent of degradation after 1 h (%)	degradation rate constant (*k*) (min^–1^)	correlation coefficient (*R*^2^)	extent of mineralization (%)
1DL-400	93.7	2.3 × 10^–3^	0.99	66.71
AN-400	95.1	2.5 × 10^–3^	0.95	
1DL-700	95.4	2.5 × 10^–3^	0.98	70.51
AN-700	95.1	2.6 × 10^–3^	0.93	

a Also listed in the last column is the extent of mineralization as measured by TOC.

From the totality of these results, we conclude that

i) Neither 1DL-Col (Figure 6A), 1DL-RT (Figure 6B), nor 1DL-300 (Figure 6C) degrade BPA if the focus is on the 276 nm peak. If the focus is on the 225 nm peak, however, the 1DL-RT samples do reduce its intensity (Figure 6B).
ii) In sharp contrast to our previous 1DL work for the degradation of cationic dyes,^79,80^ there is little adsorption of BPA onto any of our samples. This is reasonable, since BPA is not a cation, which is an important requirement for adsorption onto unmodified 1DLs.^78^
iii) For temperatures of 400 °C and higher, the annealing temperatures have a small effect on the degradation (Figure 7A). Annealing at 700 °C, however, slightly enhances BPA degradation (Figure 7A).
iv) The absorption spectra for the 1DL-400, -600, and -700 samples shown in Figure 6D–F, respectively, make it amply clear they effectively degrade BPA under a one sun simulated spectrum. It follows that the best performing polymorph of titania in degrading BPA is anatase.^113,114^
v) The AN powders degrade BPA slightly better than the 1DLs PMPs (Figure 7B–D). The extent of degradation after 1 h is tabulated in Table 3.
vi) For the most part, the AN and P25 powders are better at degrading BPA than the annealed 1DLs (Figure S10). This is especially true after the P25 powder is annealed at 400 °C (Figure S11). Interestingly, the annealed P25 and AN powders degrade BPA faster than their as-received counterparts (P25-RT and AN-RT in Figure S11).
vii) Based on the results shown in Figure 8 and Table 3, it is reasonable to assume the kinetics are indeed linear with rate constants that are weak function of annealing times and/or the anatase source. The photodegradation experimental data fits quite well with the zero-order kinetics (Figure 8). The fit appears linear after a slight delay.
viii) Lastly, we also explored whether changes in pH had any effect on BPA degradation by 1DL-RT. In one experiment, we adjusted the pH of the initial solution—at the beginning of the experiment by adding 0.1 M NaOH—to increase the pH to 11.2. No adsorption or degradation was observed (Figure S12). There was a shift in light absorption behavior, presumably due to the pH change.

**Figure 9:**
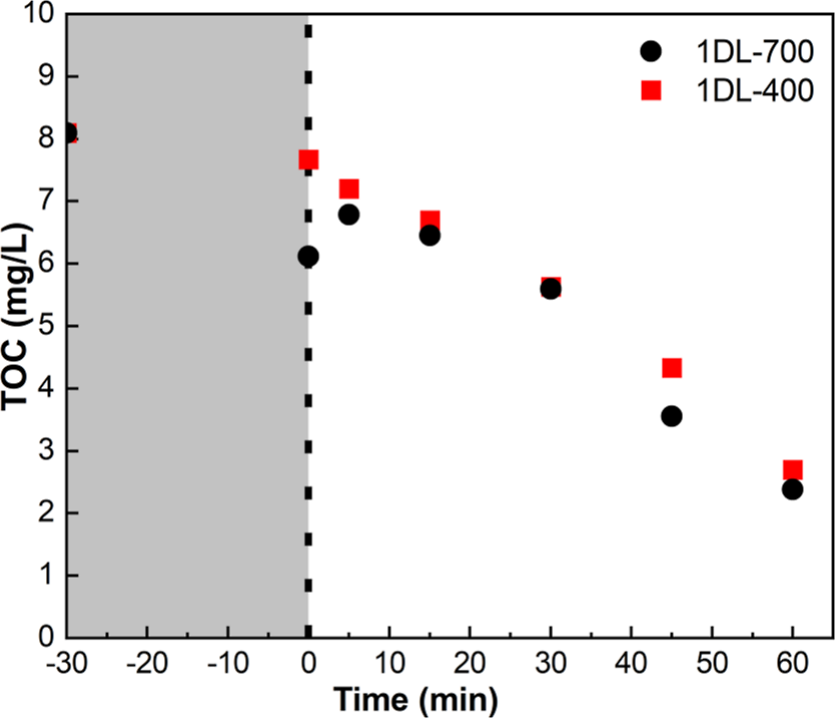
Time dependence of TOC in solutions following photodegradation by 1DL-400 and 1DL-700 powders. Extrapolating lines passing through data points suggest total degradation would occur in ≈90 min. Note this also includes any TMA molecules present.

To confirm that we are indeed degrading BPA and not simply converting it to other smaller organic molecules, we measured the time dependence of the TOC. The result (Figure 9) shows that TOC reduced from 8.1 to 2.7 mg/L for the degradation of BPA by 1DL-400 and to 2.4 mg/L BPA by 1DL-700 in 1 h. 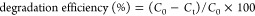
2The extents of degradation calculated from eq 2 are 66.7% and 71% for 1DL-400 and 1DL-700 powders, respectively (Figure S13). The breakdown of organic carbon into inorganic carbon implies that the former undergoes mineralization during the photocatalytic process of degradation.

While no mechanistic studies were carried out here, there is ample work on how anatase degrades dyes and organic molecules in the literature.^103^ There is no reason to believe that these mechanisms are not occurring here. In our case, we assume that the mechanism of BPA degradation starts with the excitation of the TiO_2_ catalyst under exposure to UV–vis light. Generated electrons in the conduction band can react with molecular oxygen adsorbed on the catalyst surface or present in water to form ^•^O_2_^–^ radicals. Photogenerated holes in the valence band react with OH^–^, water or can oxidize an organic pollutant to form ·OH radicals. Presumably, it is these reactive oxygen species that attack BPA and degrade it into environmentally safe products.^115^

## 3 Conclusions

Herein, we convert 1DL NFs, in the form of PMPs, to anatase as a function of annealing temperatures in air for 12 h. A temperature of 400 °C or higher was needed to convert the PMPs to anatase while preserving the PMP morphology. And while the former did not degrade BPA, anatase did. BPA was effectively degraded by 95% in 1 h under 1 sun of a simulated solar spectrum. Under the same conditions, the TOC analysis indicated that the organic content was reduced by 70% over the same time interval.

And while these values are not as good as commercially available P25 powders, for example, the advantages of using our material are several and include lower production costs and easier handling due to the shape of the particles. Lastly, the availability of anatase particles with all the advantages of the nanoscale but none of the downsides like agglomeration should prove to be quite advantageous in many other applications where nanoanatase is useful.

## 4 Methods

### 4.1 Materials

Titanium diboride (TiB_2_) powder (Thermo Scientific, ∼325 mesh, MA, USA), tetramethylammonium hydroxideaqueous solution, TMAOH (Alfa Aesar, 25 wt % in deionized (DI) water, 99.999%, MA, USA), BPA (Sigma-Aldrich, purity >98.9%, WI, USA), and ethanol (Decon Laboratories Inc., 200 Proof, PA, USA) are the chemicals used in the present work. All chemicals were used as received.

To compare our results with other commercially available materials, we also purchased anatase nanopowders (Thermo Scientific Chemicals, 99.7%, MA, USA) and P25 TiO_2_ (Evonik Aeroxide Acros Organics, purity >99.5%, MA, USA). These are referred to as **AN** and **P25**, respectively, in the manuscript.

### 4.2 Material Synthesis

The TiB_2_ powders were reacted with TMAOH aqueous solution for 5 days at 80 °C in a temperature-controlled shaking incubator (Labnet 211DS, 49L, 120 V, Woodbridge, NJ, USA). The Ti:TMAOH mole ratio was maintained at 0.6 (viz., 7 g TiB_2_ to 70 mL TMAOH) to allow us to compare the results obtained here with our previous work.^72^ This ratio is far from being optimized. The reaction solution was allowed to cool and was washed several times (typically 3 times with 50 mL of ethanol) with ethanol until a pH ≈ 7 is obtained. The supernatant was then decanted and discarded after each step. The resulting powders were dehydrated overnight in open air at 50 °C to obtain 1DL PMPs. 1DL colloids (1DL-Col) were prepared by adding DI water to the ethanol-washed product followed by centrifugation for 60 min at 5000 rpm to remove any unreacted TiB_2_ precursor. Note water needs to be added to the powders before they dry.

The obtained 1DL PMPs were annealed in air at 200 °C, 300 °C, 400 °C, 600°C, or 700 °C for 12 h in a furnace for the majority of the samples in this study. Some were also heated to 800 °C for 12 h, but we saw a mixed phase anatase/rutile product. In addition to annealing our materials, we also heated the AN and P25 powders to 400 or 700 °C for 12 h. The sample designations and annealing temperatures are given in Table 1.

### 4.3 Material Characterization

Crystal phase identification and crystallite size were obtained from powder XRD patterns carried out at room temperature, RT, using a diffractometer (Rigaku Miniflex 600). X-rays with Cu κ_α_ radiation were used. The step size was 0.04°, and the duration time per step was 1 s.

Samples were imaged in a field emission SEM (Supra 50VP, Carl Zeiss Ag, Jena, DE) and a field emission TEM (JEOL 2100F, JEOL Ltd., Tokyo, JP). TEM samples were prepared by drop casting onto a lacey carbon grid, using isopropanol as the dispersant. Samples were sputter coated for 30 s at 40 mV with a Pt/Pd sputter coater (208HR, Cressington Scientific Instruments, Watford, UK) prior to being placed in the scanning electron microscope.

FTIR spectra of the powders were obtained at a resolution of 4 cm^–1^ in the 400 to 4000 cm^–1^ range (INVENIO R with attenuated total reflection (ATR) attachment, Bruker Corp., Billerica, MA, USA). The untreated powders were placed directly on the ATR crystal, and pressure was applied by twisting down the hammer. The raw data were corrected using a standard ATR correction.

A spectrometer (VersaProbe 5000, Physical Electronics, Chanhassen, MN, USA) was used to obtain XPS spectra. The samples were analyzed without Ar^+^ sputtering. The samples were mounted on an Al stub via carbon tape. Monochromatic Al K_α_ X-rays, with a pass energy of 27.0 eV, a step size of 0.50 eV, 0.1 s step time, and a spot size of 200 μm, were used to irradiate the sample surfaces. Five scans were obtained for each region. The CasaXPS version 2.3.23PR1.0 software was used for analysis. The obtained spectra were calibrated by setting the C–C bond to 285.0 eV.

### 4.4 Photocatalysis Experiments

The photocatalytic activities of all powders were studied by monitoring the BPA decomposition in water. Prior to the photodegradation, a mixture of the catalyst and BPA solutions (unless otherwise noted, 3.5 mg of dried 1DL PMPs and 20 mL BPA (10 mg/L)) was stirred at 500 rpm for 30 min in the dark to achieve adsorption equilibrium. The initial pH of the BPA solution was 6.3 and remained unadjusted, unless otherwise noted.

The photodegradation of BPA was monitored with a UV–vis spectrophotometer (Cary 60, Agilent, Santa Clara, CA, USA) in the 200–500 nm range, with a scan rate of 60 nm/min in quartz cuvettes. The process is shown schematically in Figure S1. To determine the BPA content in the solutions, we focused on the λ_max_ = 276 nm peak, characteristic of BPA.

Photodegradation experiments utilized a 300 W xenon solar simulator equipped with a UV–vis mirror module, quartz light guide, and 1.0× downward type collimator lens (MAX-DRLQ1-B MAX 350, Asahi Spectra, Tokyo, Japan). A constant output irradiance of 100 mW/cm^2^ was shined on the catalyst-BPA suspension interface. Prior to each experiment, irradiance was measured using a thermal power sensor (S401C with a PM400 console, Thorlabs, Newton, NJ, USA). To keep the catalyst suspended, we continually stirred the solution with a 10 mm PTFE-coated magnetic stir bar. Aliquots of 3 mL were taken out at prespecified times and filtered through a 0.22 μm PTFE syringe filter made of hydrophobic polypropylene (Thermo Fisher Scientific, Carlsbad, CA, USA). Degradation efficiency is calculated based on eq 2where *C*_0_ is the initial concentration of the BPA aqueous solution and *C*_*t*_ is the concentration of BPA at time *t*.

### 4.5 TOC Analysis

TOC in the form of nonpurgeable organic carbon (NPOC) was measured using a TOC analyzer (Aurora 1030w, 1088 autosampler, OI Analytical, College Station, TX, USA). Samples were diluted with water 20 times and transferred to 40 mL borosilicate glass vials (VWR TraceClean clear vials), which had been cleaned using a 20% HCl rinse followed by a triple water rinse. The vials containing the solution were heated in a muffle furnace at 500 °C for 4 h. NPOC was measured in accordance with Standard Method 5310C and EPA 415.3 by acidifying 3.0 mL aliquots of sample in a heated 98 °C glass reaction vessel with 2.0 mL of phosphoric acid (5% w/v) and sparging for 2 min with compressed air (Airgas Ultra Zero grade air) to remove purgeable organic and inorganic carbon. Sodium persulfate (10% w/v) was then introduced to the sample, oxidizing the remaining carbon into CO_2_, which was detected by a nondispersive infrared detector and reported in mass and concentration units. All samples were measured in duplicate, and internal lab duplicates were performed every 5 samples.

The system is calibrated using a range of aqueous solutions of potassium hydrogen phthalate), diluted from a stock NIST-traceable standard solution (Supelco Certipur TOC standard solution). The calibration range for these analyses ranged from 0.5 to 7.0 mg/L TOC. Calibration accuracy is verified by analyzing separate QC check solutions (3.5 mg/L and 7.0 mg/L antipyrine, Sigma) and lab fortified blanks (0.1 mL 700 mg/L antipyrine in 20 mL DI water). The detection limit for solutions is <10 ppb.

## Supporting Information Available

Figures detailing experimental setup, additional XRD analysis, all XPS analysis, additional SEM micrographs, additional TEM micrographs and SAED pattern, additional solid-state optical measurements, additional UV–vis results of BPA degradation, and additional TOC conclusions (PDF)
