# Supplementary material for: Hierarchically Porous Anatase Nanoparticles Derived from One-Dimensional Lepidocrocite Titanate for Bisphenol-A Photodegradation
Source: ACS Omega. 2024 Dec 11;10(5):4406–17. doi: 10.1021/acsomega.4c07224 (PMC11822498; doi:10.1021/acsomega.4c07224)
Supplement: Supplementary file 1 — ao4c07224_si_001.pdf [file ao4c07224_si_001.pdf]

Supporting Information

Hierarchically Porous Anatase Nanoparticles Derived from One-Dimensional Lepidocrocite Titanate for Bisphenol-A Photodegradation

Treesa Reji[1], Adam D. Walter[1], Yasunori Hioki[2], Tracey Curran[3], Mary Qin Hassig[1], Hussein O. Badr[1], Gregory R. Schwenk[1], Takeshi Torita[2] Megan Creighton[4], and Michel W. Barsoum[1,*]

[1]Department of Materials Science and Engineering, Drexel University, Philadelphia, PA

[2]MuRata Manufacturing Co., Ltd, Nagaokakyo-shi, Kyoto, Japan

[3]Academy of Natural Sciences of Drexel University, Philadelphia, PA

[4]Department of Chemical and Biological Engineering, Drexel University, Philadelphia, PA

*Corresponding Author (barsoumw@drexel.edu)

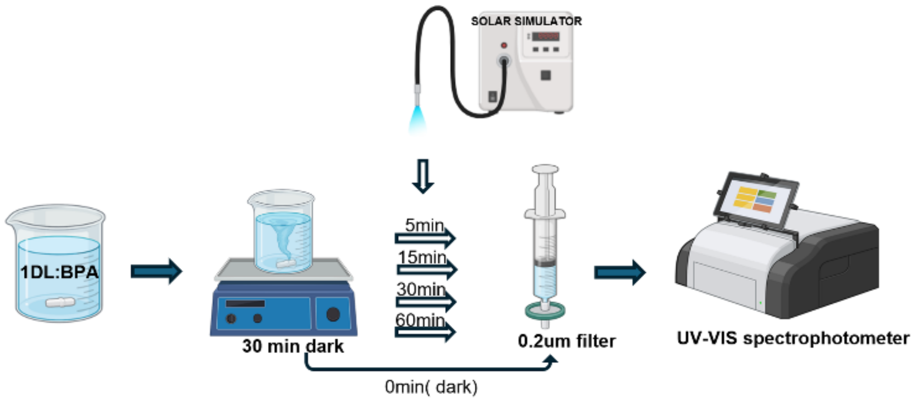

Figure S1. Schematic depicting procedure to study the photodegradation of BPA by 1DL powders tested herein. In all cases, the mixtures were filtered through a 0.2 µm filter before measuring their optical properties

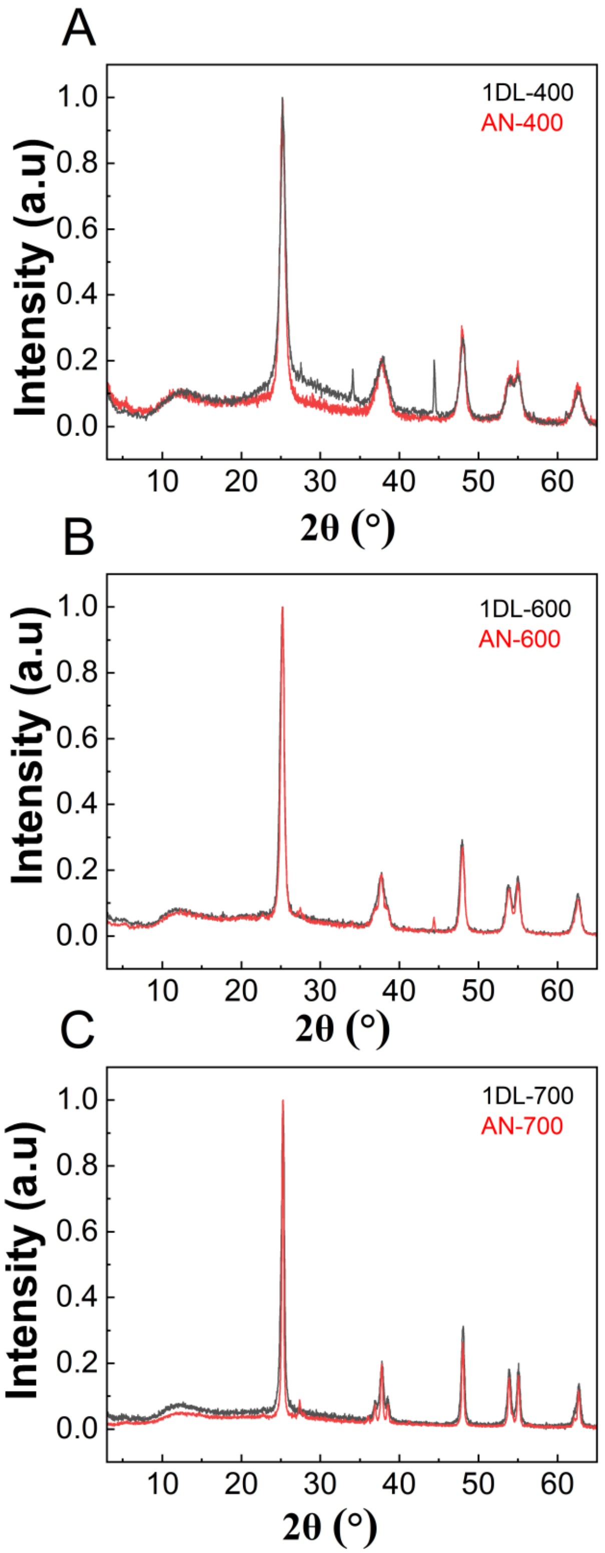

Figure S2. Indexed XRD patterns comparing 1DL and AN powders annealed at, A) 400 °C, B) 600 °C, and C) 700℃. Labels and patterns are color coordinated.

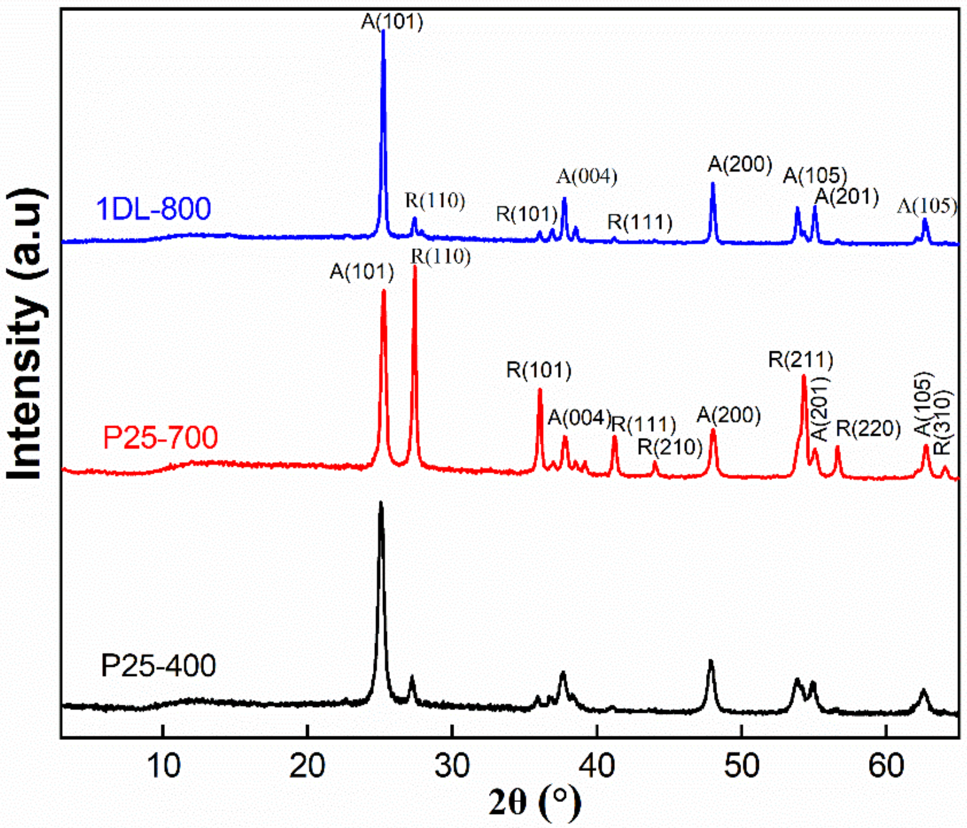

Figure S3. Comparison of XRD patterns of 1DLs annealed at 800 ℃ for 12 h (blue), and P25 annealed at 400 °C (black) and 700 °C (red). Labels and patterns are color coordinated.

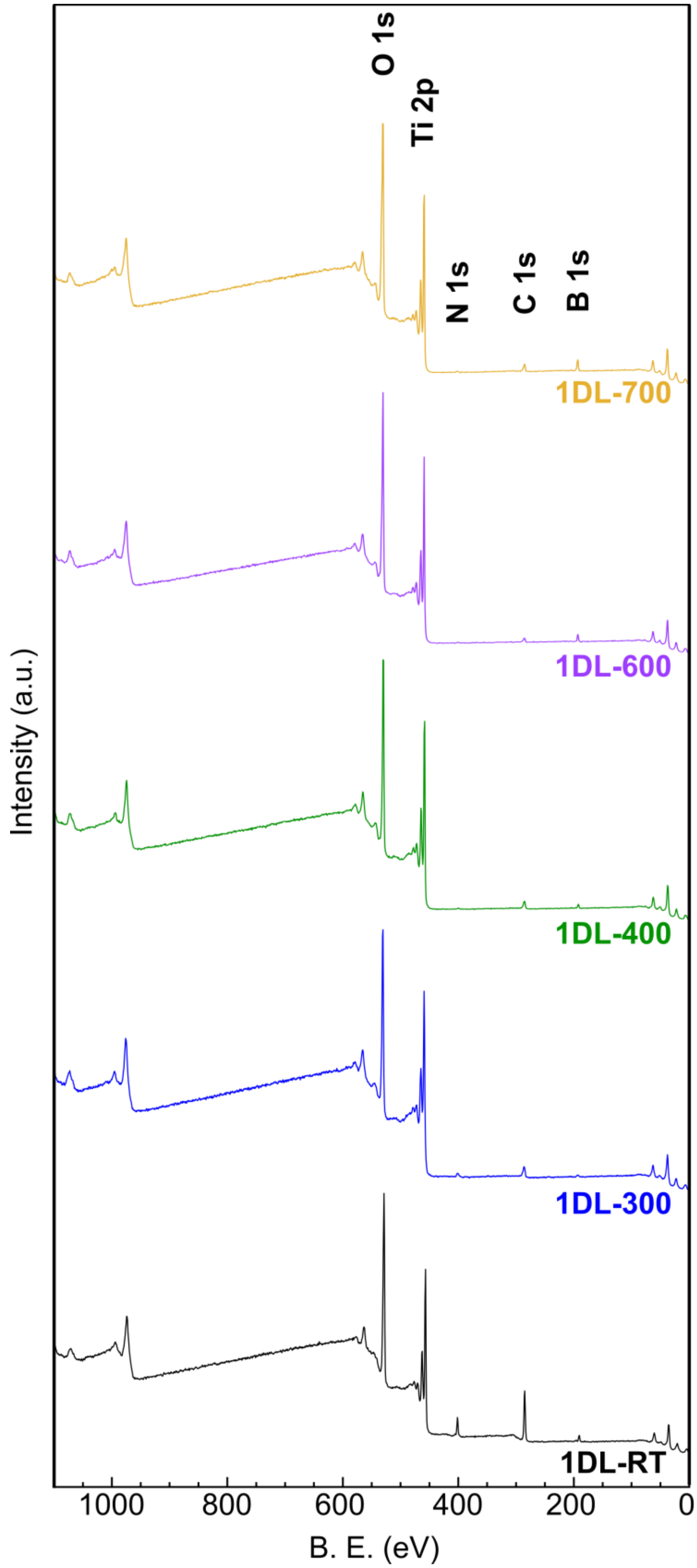

Figure S4. X-ray photoelectron spectroscopy (XPS) survey spectra of 1DL-anatase samples. Labels and spectra are color coordinated.

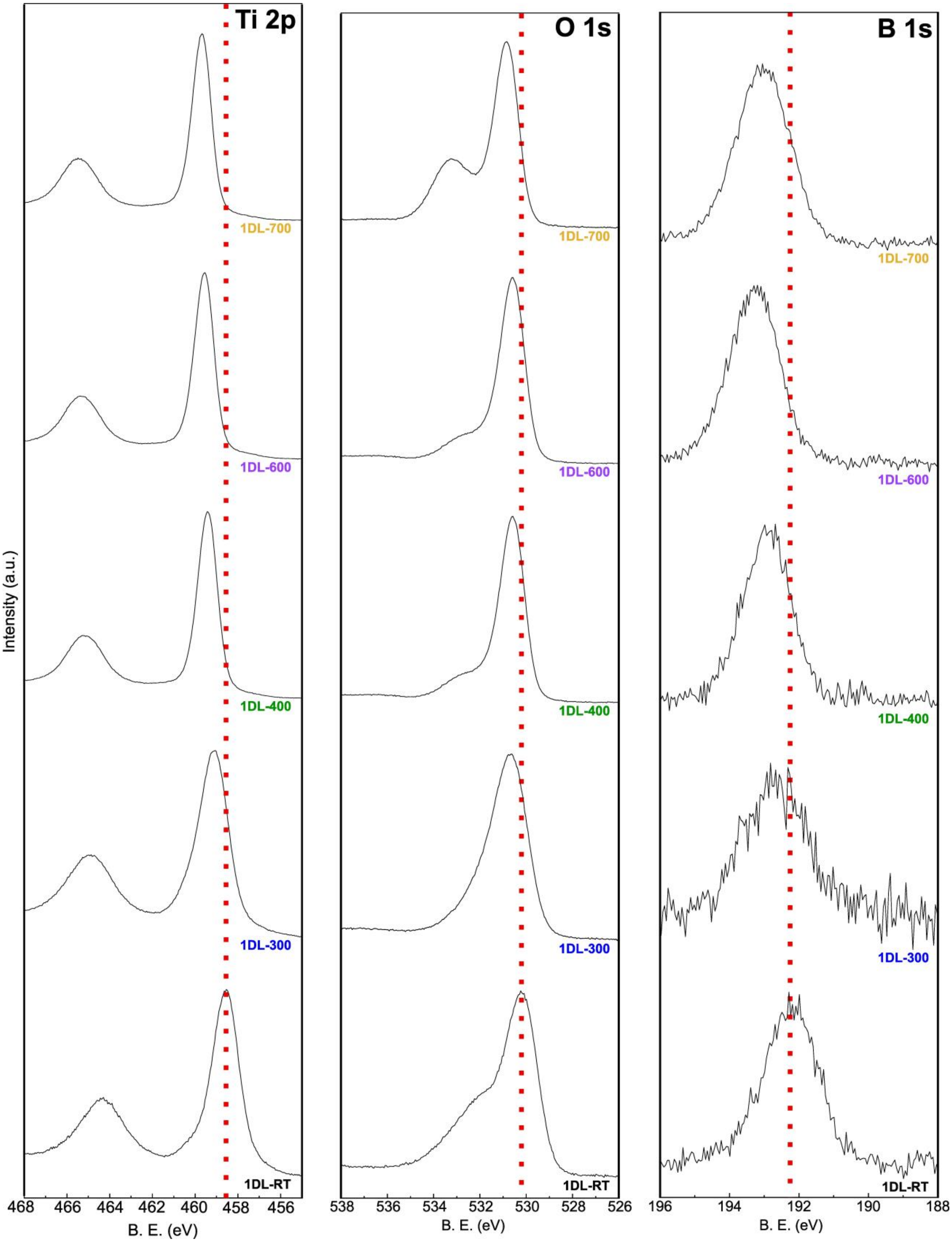

Figure S5. XPS element spectra of 1DL-anatase samples for Ti 2p, O 1s, and B 1s regions. Labels are color coordinated.

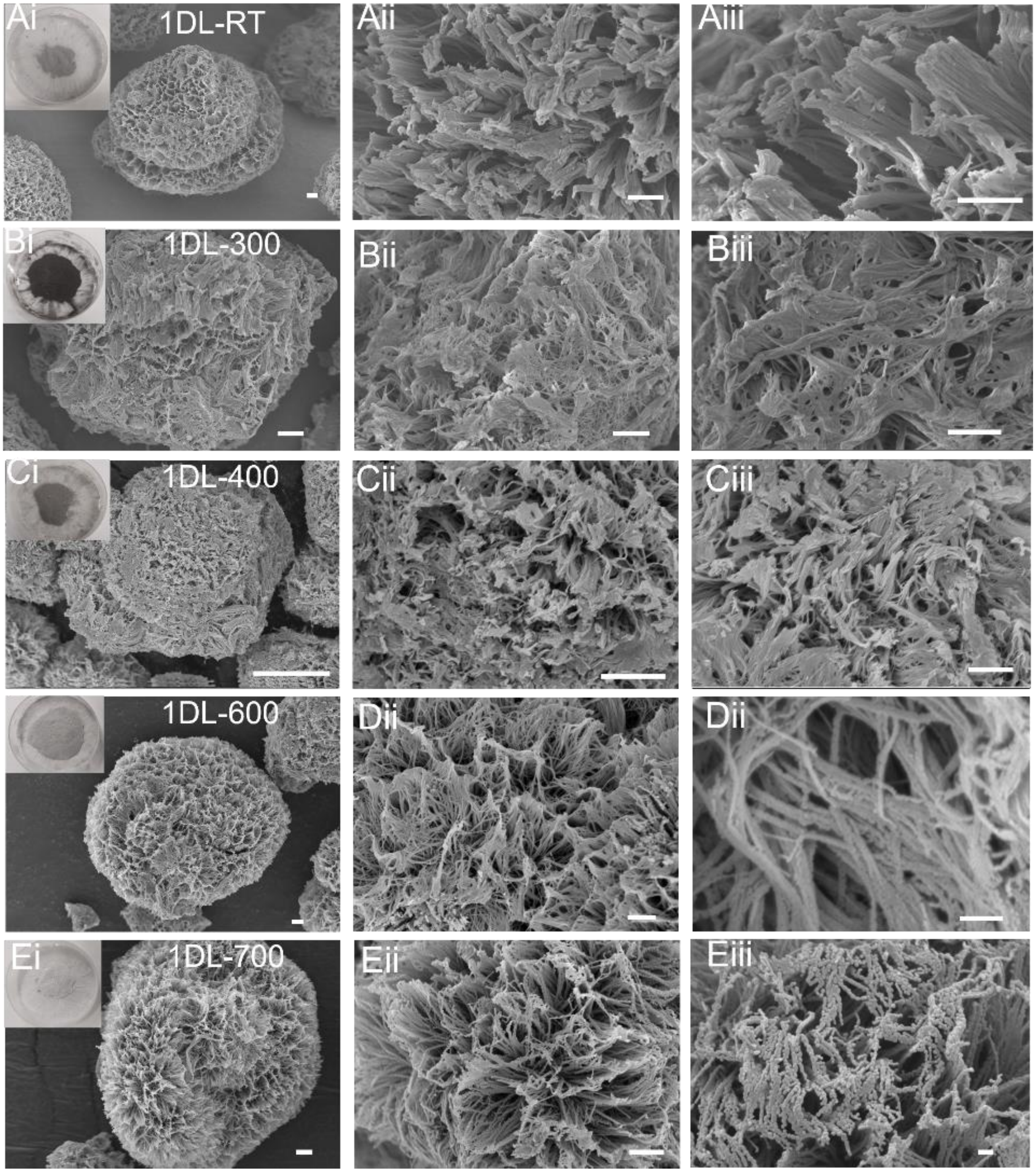

Figure S6. SEM micrographs of 1DL PMPs as a function of annealing tempearatures for 12 h. (Ai-Aiii): 1DL-RT; (Bi-Biii), 1DL-300; (Ci-Ciii), 1DL-400; (Di-Diii), 1DL-600 and (Ei-Eiii), 1DL-700. Insets in column i are photographs of each powder after annealing. **Scale bars:** Column i, 2 μm; Column ii, 1 μm; Column iii, 200 nm.

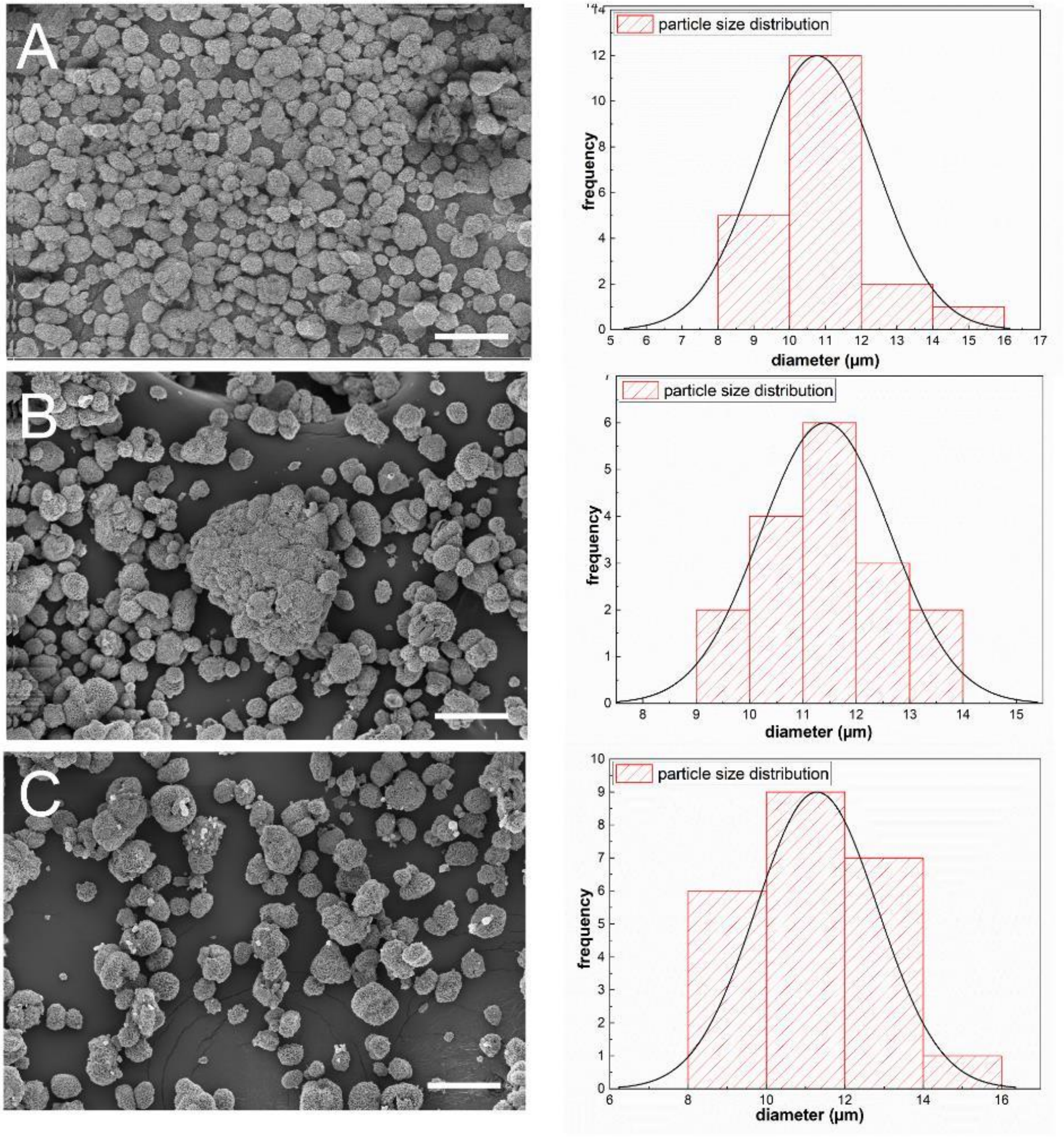

Figure S7. Low magnification SEM micrographs for, A) 1DL-400, B) 1DL-600, C)1DL-700 powders, with corresponding particle size distribution plots on the right. Scale bar = 10 μm.

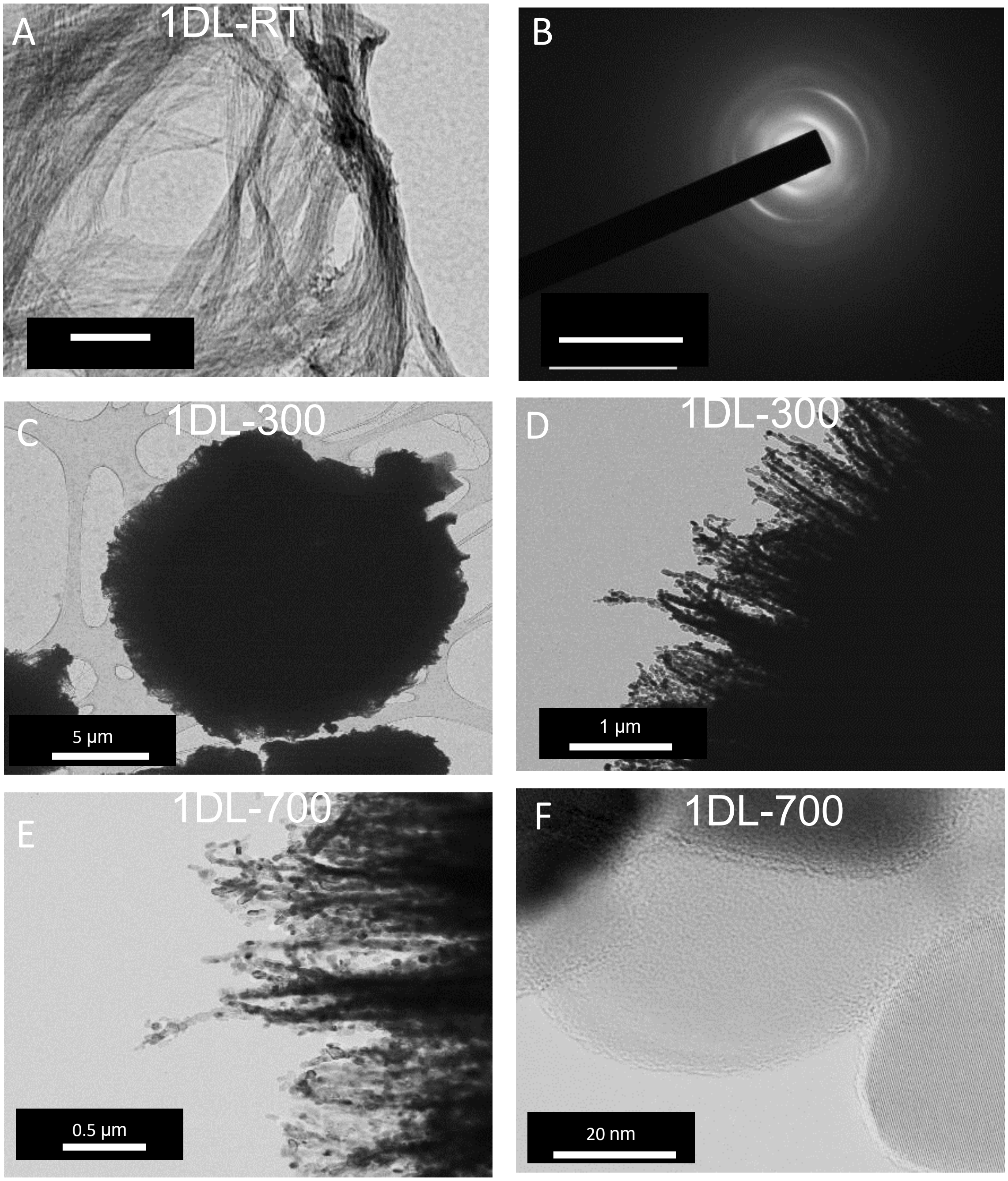

Figure S8. Typical TEM micrographs for, (A), 1DL-RT; (C), 1DL-300; (D-F), 1DL-700 samples. B) shows SAED of micrograph shown in A, showing characteristic arcs indicative of a fiber structure.

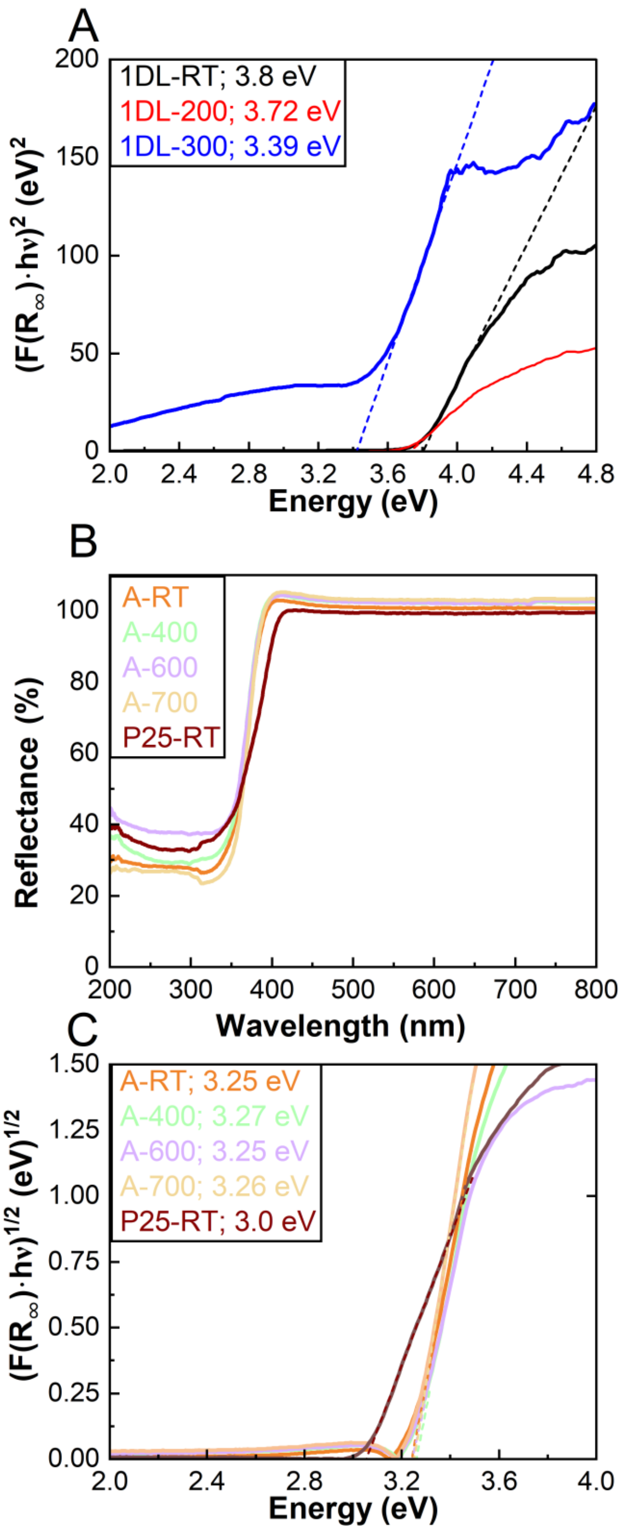

Figure S9. Optical properties of select powders measured. A) Tauc plot for direct transition of 1DL-RT (Black), 1DL-200 (Red), 1DL-300 (Blue). B) UV-Vis reflectance spectra of AN powders as a function of annealing temperature and that of P25; C) Tauc plot of same results. Labels and patterns are color coordinated.

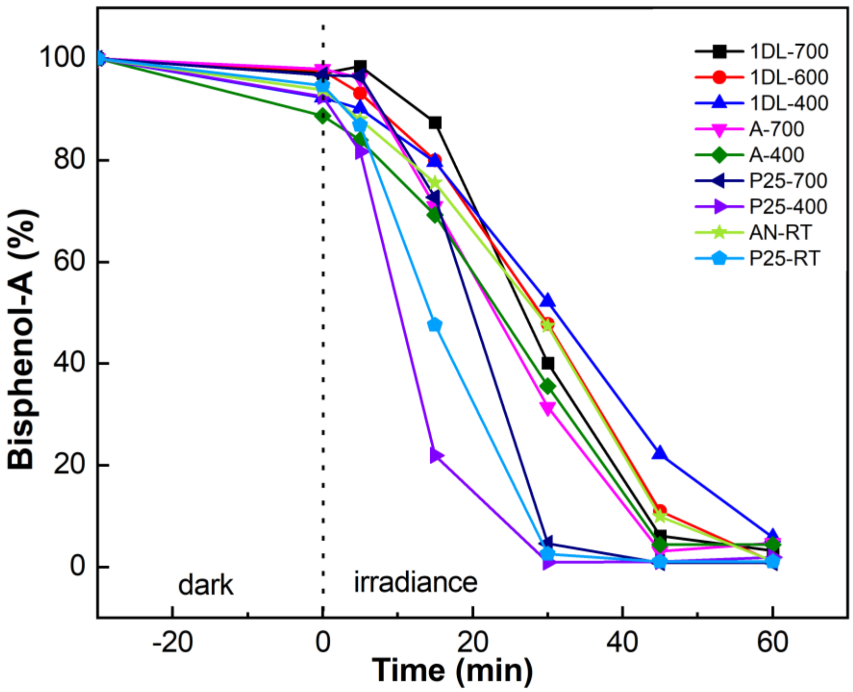

Figure S10. Relative concentrations of BPA as a function of photocatalyst. Labels and patterns are color coordinated.

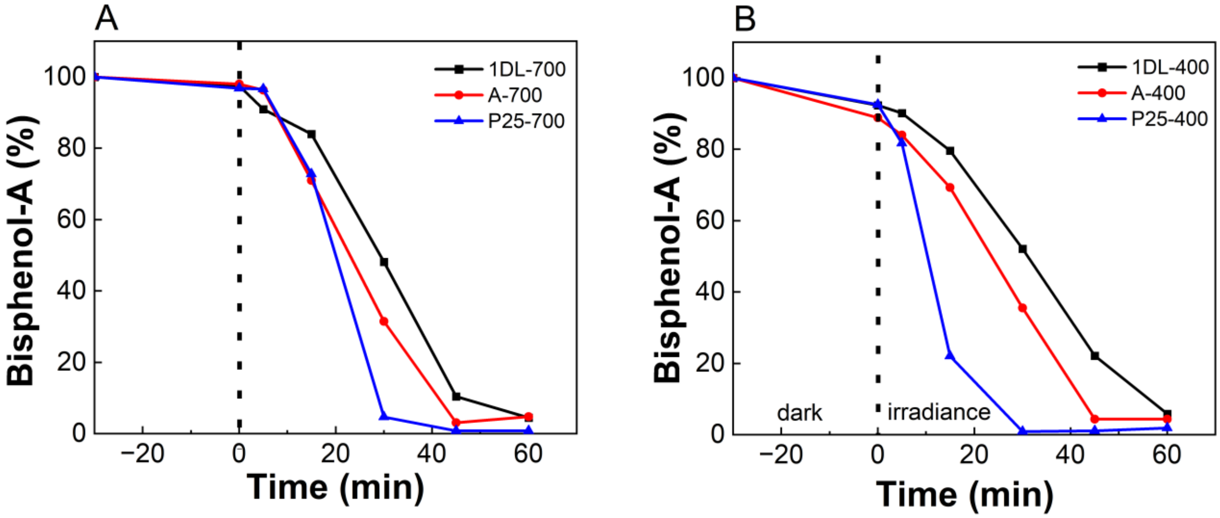

Figure S11. Relative concentration of BPA as a function of catalysts annealed at, A) 400℃ and (B) 700℃. Labels and patterns are color coordinated.

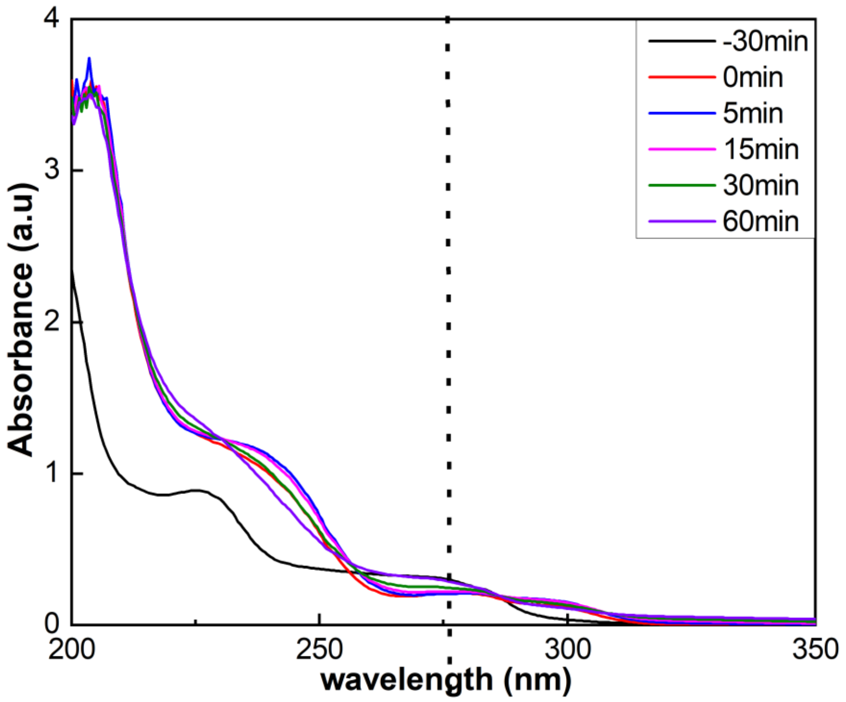

Figure S12. Time dependence of BPA (10 mg/L) degradation by, 1DL-RT sample with the initial pH of the experiment set to 11.2. Vertical dashed lines correspond to λ = 276 nm which is used to follow the BPA degradation.

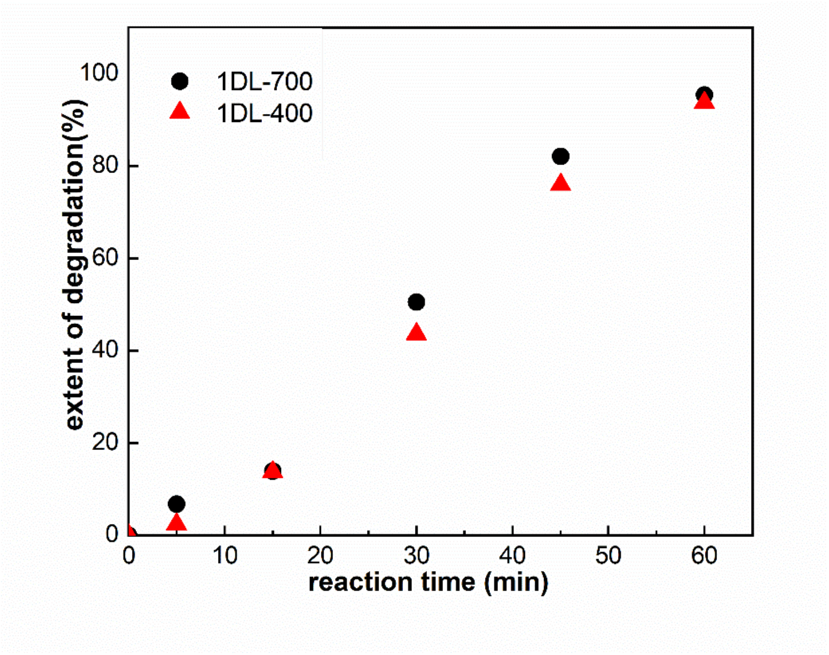

Figure S13. Extent of BPA degradation by 1DL TiO_2 derived anatase after annealing at 400℃ and 700℃ for 12 h. Annealing temperature does not affect the extent of degradation much.
